# Body condition of dogs fed diets containing soya hulls*

**DOI:** 10.1017/jns.2014.45

**Published:** 2014-09-30

**Authors:** Tabyta T. Sabchuk, Mariana Scheraiber, Carolina P. Zanatta, Alex Maiorka, Ananda P. Félix, Simone G. Oliveira

**Affiliations:** Animal Science Department, Universidade Federal do Paraná, Rua dos Funcionários, 1540, Curitiba 80035-050, PR, Brazil

**Keywords:** Canine nutrition, Fibre sources, Obesity, 0SH, 0 % soya hulls, 16SH, 16 % soya hulls, BCS, body condition score, BF, body fat, CBMI, canine BMI, BW, body weight, ME, metabolisable energy, MER, metabolisable energy requirements

## Abstract

Obesity is a growing problem in dogs. Therefore, there is an increasing need of foods for obese dogs with high-fibre content to dilute energies and to reduce energy absorption. Soya hulls are cheap and are widely available as a fibre source. We aimed at evaluating the body condition of dogs fed diets containing 0 % soya hulls (0SH) or 16 % soya hulls (16SH) in replacement of maize. Twelve adult dogs, with 11·3 (se 1·6) kg average body weight (BW), 4·1 (se 0·1) years old and body condition score (BCS) between 4 and 7, were completely randomised assigned (six per treatment) and were fed the 0SH diet according to their maintenance energy requirements or the same amount in grams (g/kg BW^0·75^) of the 16SH diet once daily for 56 d. The animals were evaluated on days 0 and 57 for BW, BCS (1, very thin to 9, obese), subcutaneous fat thickness in the L7 vertebra using ultrasound (L7), canine BMI (CBMI) and body fat (BF). Data were analysed by the Student's *t* test and Kruskal–Wallis test (*P* < 0·05). The change (final – initial) in BW (−0·58 *v.* −0·49 kg), BCS (−1 *v.* −1), L7 (−2 *v.* 0·35 mm), CBMI (−0·85 *v.* −0·63 kg/m^2^) and BF (−5·0 *v.* −5·4 %) of dogs fed the 0SH and 16SH diets, respectively, were not different (*P* > 0·05). The 16SH diet, with 11·4 % restriction in metabolisable energy, did not change the BCS of adult dogs. Further studies evaluating the supply of soya hulls only to overweight/obese dogs should to be carried out, because these dogs may respond differently than the group evaluated, which had a BCS between 4 and 7 (ideal to overweight).

Interestingly, studies in several regions of the world estimate that 22–40 % of the dog population is obese or overweight^(^^1^^)^. Genetics may be an underlying cause of obesity, as some breeds are more predisposed than other to be obese or overweight. In addition, very palatable diets may cause dogs to ingest more energy than they need. Furthermore, the lack in food supply control by the owners and sedentary lifestyles^(^^2^^)^ also contribute to dog obesity. Overweight and obese dogs may suffer health problems, including orthopaedic and respiratory conditions, diabetes mellitus and other diseases^(^^3^^)^.

Therefore, several methods have been attempted to prevent and to mitigate obesity in dogs. According to German^(^^3^^)^, these include changing dog's lifestyle by increasing physical activity, monitoring dog's weight and dietary energy restriction, which is one of the most efficient practices to make dogs lose weight. Energy intake can be reduced by supplying food in quantities below the requirements or by including fibre sources to the diet.

Dietary fibre may reduce body fat (BF), slow gastric emptying, lower blood lipids and glucose concentrations as well as insulin concentrations^(^^4^^)^, energy digestibility^(^^5^^)^ and enhance satiety^(^^6^^)^. However, these effects depend on the physical–chemical characteristics of the fibre source and its inclusion level in the diet.

Soya hulls, the fibre source used in the present study, contains mainly insoluble fibre (insoluble-to-soluble fibre ratio between 15·4:1 and 5·0:1^(^^5^^)^). It is widely available in the market and may reduce costs for the pet food industry. Therefore, the present study aimed at evaluating the body condition of dogs fed diets containing or not soya hulls.

## Materials and methods

The experiment was approved by the Committee of Ethics on Animal Use of the sector of Agrarian Sciences of the Federal University of Paraná, Curitiba, PR, Brazil, under protocol no. 019/2012.

### Animals and facilities

Twelve adult beagles (six males and six females), with 11·3 (se 1·6) kg average body weight (BW), with body condition score (BCS) between 4 and 7, and 4·1 (se 0·1) years of age, were studied. All dogs were previously submitted to clinical and physical examinations, vaccinated and dewormed. Dogs were individually housed in concrete kennels with solarium (5 m long × 2 m wide).

### Diets

Soya hulls were included in the diet at the expense of maize. Two diets were formulated: without soya hulls (0SH) or with 16 % soya hulls (16SH), as shown in Table 1. The diets were ground in a mill using 1·0 mm mesh, and were extruded in a single-screw extruder (E-130; Ferraz). Diets were analysed for DM, crude protein, diethyl ether extract in acid hydrolysis, crude fibre, ash, calcium and phosphorus contents, according to the AOAC^(^^7^^)^. Dietary contents of total dietary fibre, soluble fibre and insoluble fibre were determined according to the methods of Prosky *et al.*^(^^8^^)^. Gross energy was determined in a bomb calorimeter (Parr Instrument Co. model 1261), and metabolisable energy (ME) was determined *in vivo* in a previous total faecal collection digestibility trial with eight adult Beagle dogs, with eight replicates per treatment (unpublished results, Tabyta Tamara Sabchuk, 2013), according to the Association of American Feed Control Officials (AAFCO)^(^^7^^)^.

**Table 1.** Ingredients and analysed chemical composition of soya hulls and of the diets without (0SH) and with soya hulls (16SH)

Ingredients (%)	Soya hulls	0SH	16SH
Maize	–	56·3	40·5
Poultry fat	–	3·0	3·0
Maize gluten	–	6·0	6·0
Poultry by-product meal	–	30·0	30·0
Salt	–	0·5	0·5
Poultry hydrolysate	–	2·0	2·0
BHA	–	0·015	0·015
BHT	–	0·015	0·015
Citric acid	–	0·05	0·05
Calcium propionate	–	0·4	0·4
Choline chloride	–	0·4	0·4
Mineral and vitamin supplement*	–	0·5	0·5
Calcium carbonate	–	0·303	0·0
Potassium chloride	–	0·514	0·577
Soya hulls	–	0·0	16·0
Total	–	100	100
Chemical composition (% of DM)			
DM	89·79	93·44	93·38
Crude protein	13·01	28·05	29·33
Diethyl ether extract in acid hydrolysis	5·88	14·84	12·8
Crude fibre	38·45	2·99	7·42
Total dietary fibre	72·08	14·4	24·98
Insoluble fibre	65·51	14·16	18·73
Soluble fibre	6·57	0·24	6·25
Insoluble fibre: soluble fibre ratio	9 : 1	59 : 1	3·0 : 1
Ash	–	7·69	7·96
Calcium	–	1·01	0·73
Total phosphorus	–	1·14	1·09
Metabolisable energy (MJ/kg)	–	17·91	15·78

*Enrichment.kg of food^−1^: Vit. A, 2000 IU; Vit. D_3_, 2000 IU; Vit. E, 480 IU; Vit. K_3_, 48 mg; Vit. B_1_, 4 mg; Vit. B_2_, 32 mg; B_12_, 0·2 mg; pantothenic acid, 16 mg; niacin, 56 mg; choline, 800 mg; zinc, 150 mg; iron, 100 mg; copper, 15 mg; iodine,1·5 mg; manganese, 30 mg; selenium, 0·2 mg and antioxidant 240 mg.

Dogs were offered the 0SH diet in sufficient amount to try supply their metabolisable energy requirements (MER; MJ/d), calculated by the equation: MER = 0·54 × BW^0·75^, according to the NRC^(^^9^^)^. The allowance of the 16SH diet was calculated as a function of the ME value of the 0SH diet. This calculation was used to restrict only energies, allowing the dogs to ingest the same volume of feed in grams of the 0SH diet. Dogs were fed once daily, at 08:00 hours. Food intake was measured daily and water was offered *ad libitum*.

### Body condition scoring

The experimental period was 57 d. Fasted BW and BCS were evaluated on the first and the last day of the experiment. BCS was determined according to the method of Laflamme^(^^10^^)^, in a 1–9 scale (1, very thin; 9, obese) and subcutaneous fat thickness (mm) was measured at L7 lumbar vertebra by ultrasound in transversal plane using a 7·5 MHz linear transducer, as proposed by Morooka *et al.*^(^^11^^)^. Canine BMI (CBMI) was calculated as proposed by Muller *et al.*^(^^12^^)^, according to the equation: CBMI = BW/height^2^. Height was measured from the base of the neck to the base of the tail.

BF % was calculated by the following equations, as proposed by Burkholder & Toll^(^^13^^)^:

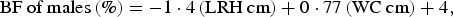


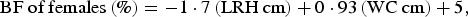

where LRH is the length of the right posterior limb, from the tuberosity of the calcaneus to the medium patellar ligament. WC is the waist circumference (from the middle point between the iliac crest and the last thoracic vertebra, measured with the dog in standing position).

The methodologies applied in the present study were based on previously published studies^(^^11^^–^^14^^)^, which demonstrated their efficacy to determine BCS.

### Statistical analysis

The differences between the measurements made at the beginning and at the end of the experimental period (end–beginning) were analysed. A completely randomised experimental design was adopted. Data were tested for normality, and then Student's *t* test was applied to parametric data and Kruskal–Wallis test for non-parametric data. In both tests, differences with *P* < 0·05 were considered significant. All analyses were carried out using SAS statistical package (Statistical Analysis System, version 8·2; SAS Inst. Inc.).

## Results

In the beginning of the experiment, dogs presented an average BW of 11·31 (se 1·59) kg and BCS between 4 and 7. Most dogs had BCS of 4–6, which are considered normal. Diet intake in grams of the diet 0SH (206·67 g) and diet 16SH (205·0 g) did not differ (*P* > 0·05), whereas ME intake was lower in dogs fed the 16SH diet (518 kJ ME/kg BW^0·75^ per d) than diet 0SH (621·86 kJ ME/kg BW^0·75^ per d) (*P* > 0·05; Table 2). The change from baseline (final–initial) in BW (−0·58 v*.* −0·49 kg), BCS (−1 *v.* −1), L7 (−2 *v.* 0·35 mm), CBMI (−0·85 *v.* −0·63 kg/m^2^) and BF (−5·0 *v.* −5·4 %) of dogs fed diets 0SH and 16SH, respectively, did not differ (*P* > 0·05; Table 2).

**Table 2.** Food intake (g), metabolisable energy intake (ME; kJ ME/kg BW^0·75^ per d), initial (0 d) and change from baseline (final–initial) in the body weight (BW; kg), body condition score (BCS), ultrasound assessment at L7 (L7; mm), canine BMI (CBMI) and body fat (BF; %) of dogs (six per treatment) fed a control diet (0SH) or a diet containing 16 % soya hulls (16SH) for 57 d

Treatment	Food intake	ME intake	BW	BCS*	L7	CBMI	BF
0SH (initial – i)	–	–	11·525	5·50	21·382	16·440	24·499
0SH (final – f)	–	–	11·075	4·50	20·845	15·725	22·108
*P*			0·321	0·124	0·258	0·247	0·152
16SH (initial)	–	–	11·465	6·5	21·441	17·922	26·148
16SH (final)	–	–	10·952	5·5	20·561	17·247	24·358
*P*	–	–	0·450	0·091	0·625	0·133	0·074
0SH (f–i)	206·67	621·86	−0·450	−1	−0·537	−0·715	−3·881
16SH (f–i)	205·00	518·44	−0·545	−1	−0·880	−0·675	−4·844
sem	4·344	16·878	0·046	–	0·548	0·071	1·045
*P*†	0·858	<0·001	0·329	0·830	0·771	0·168	0·708

*BCS, 1 (very thin) to 9 (obese) scale; sem, standard error of the mean.

†Differences are significant when *P* < 0·05 between means by the Student's *t* test and between medians by the Kruskal–Wallis test (BCS).

## Discussion

The prevention and reduction of pet obesity through the diet has been widely studied^(^^4^^,^^13^^,^^14^^)^. According to Borne *et al.*^(^^4^^)^, the most effective method for weight control and weight loss is energy restriction, which can be achieved by diluting dietary energy by the use of fibres and/or by reducing food allowance.

In the present study, the diet 16SH was calculated to restrict only energies to allow the same amount of food intake (in g) as the dogs that were fed the 0SH diet. In a previous study (unpublished results, Tabyta Tamara Sabchuk, 2013), food allowance was 50 % in excess of the MER, but dogs did not stop eating when their ME requirements were met, but only when the physical capacity of their gastrointestinal tract was exceeded.

Several authors evaluated the effect of dietary fibre inclusion on the satiety of dogs. However, results were contradictory^(^^15^^)^, because the protocol to assess satiety in dogs is not well defined. Palumbo^(^^15^^)^ found that the dietary inclusion of fibre did not promote satiety in dogs. Bosch *et al.*^(^^6^^)^, on the other hand, observed that dogs fed a diet with high-fibre content presented lower intake than a diet offered 6 h after the morning meal, suggesting that fibre may be related with enhanced satiety.

Another important issue is the perception of the person responsible for the dog, who may be concerned with the dog's wellbeing, believing that the dog may be hungry if a smaller amount of food is supplied. Therefore, the inclusion of fibre in the diet dilutes dietary energy, but does not change food allowance, and consequently the dog will be fed the same amount it is used to. This effect was observed in the present study as the dogs fed the diet with high-fibre content (16SH) had lower energy intake, but the same amount of food intake (in g) as those fed the 0SH diet, as shown in Table 2. Consistent results were observed by Jewell *et al.*^(^^16^^)^, i.e. lower energy intake in dogs fed a high-fibre diet (19·4 % CF) compared with a low-fibre diet (1·7 % CF).

Despite using two different methods of energy restriction by means of dietary fibre inclusion (diets with soluble and insoluble fibres) or food restriction (45 % of the MER), Butterwick & Markwell^(^^14^^)^ did not find any effect on dog BCS. However, Fritsch *et al.*^(^^17^^)^ found that dogs lost weight faster when fed a high-fibre diet, as well as Borne *et al.*^(^^4^^)^, who verified that dogs fed a high-fibre (26·5 % CF) and low-fat (7·0 %) diet restricted to 60 % of the ME requirements lost more weight than those fed a low-fibre (2·9 % CF) and high-fat (17·9 %) diet.

In the present study, the inclusion of soya hulls did not change the BCS of the dogs during the 8-week period, whereas Borne *et al.*^(^^4^^)^, using dogs between 11·8 and 21·7 kg, observed changes in body condition when dogs were fed a high-fibre diet for 7 weeks. Therefore, it seems that time was not a limiting factor in the present experiment. However, several other factors may influence BCS assessment in dogs. In addition to evaluation time, dog nutritional status if they are obese or overweight, and age should also be considered, and may render different results. Therefore, caution must be taken when interpreting the results of studies evaluating fibre sources for dogs. For instance, Fritsch *et al.*^(^^17^^)^ observed that older dogs lost weight slower than young dogs. Also, overweight or obese dogs lose weight easier than those with normal BW because they have more BF to mobilise to compensate energy intake deficits.

The BCS of most dogs in the present study was considered normal, according to Laflamme^(^^10^^)^ (average BCS: 6), which was an important limitation, in addition to the small number of animals evaluated. However, it was not possible to use obese dogs because it is not ethical to fatten experimental dogs. Another possibility was to use naturally obese dogs, but these are not habituated to experimental conditions and their owners may interfere with their individual management. Therefore, the obtained results can only be applied to dogs with normal BCS. It is suggested that further studies on the inclusion of soyabean hulls in dog foods be performed with obese dogs. However, despite the lack of statistical difference in dogs BW at the end of the experimental period, there was a numerical reduction in those fed according to the MER of the NRC. The equation of the NRC calculates dogs' MER in general, and does not take into account the effect of each individual, i.e. its actual MER. Therefore, it is suggested to apply the equation only to calculate initial food allowance, subsequently adjusting it if dogs' weight changes.

Moreover, further energy restriction, either through dilution or restricted allowance, are probably required to obtain weight reduction, considering that the diet with soya hulls contained only 11·0 % less ME than the diet without soya hulls.

## Conclusions

Dietary energy reduction by including soya hulls in the diet did not change the dogs' BCS. However, further studies on the effect of dietary fibre on dog obesity, considering specially duration of supply and food allowance, are needed.
